# Update to the CDC influenza A(H3) subtyping assay for improved detection of influenza A(H3N2) viruses, including subclade K viruses

**DOI:** 10.1128/jcm.00727-26

**Published:** 2026-10-27

**Authors:** Bo Shu, Jimma Liddell, Phili Wong, Eduardo Salinas Duron, Ji Liu, Norman Hassell, Juliana DaSilva, Kai-Hui Wu, Angie Foust, Rebecca J. Kondor, C. Todd Davis, Marie K. Kirby

**Affiliations:** 1 Influenza Division, Centers for Disease Control and Prevention 1242, Atlanta, Georgia, USA; 2 Chippewa Government Solutions, Sault Tribe Incorporated, Sault Sainte Marie, Ontario, USA; Vanderbilt University Medical Center, Nashville, Tennessee, USA

**Keywords:** influenza subtyping, subclade K viruses, influenza A(H3) viruses

## Abstract

Influenza A(H3N2) viruses circulate globally and cause respiratory illness. The FDA-cleared CDC Human Influenza Virus Real-Time RT-PCR Influenza A (H3/H1pdm09) Subtyping assay is used to subtype seasonal influenza A viruses. However, emerging mutations in the H3 reverse primer region raised concerns about reduced assay sensitivity for contemporary circulating viruses. We updated the H3 assay by redesigning the reverse primer to target a more conserved region of the hemagglutinin gene, and incorporating minor adjustments into the forward primer and probe. Analytical sensitivity, inclusivity, and exclusivity evaluations were performed using cultured influenza viruses, synthetic RNA, and panels of non-influenza respiratory pathogens. Clinical performance was evaluated using seasonal A(H3N2) residual respiratory specimens. The updated assay (H3_v2) demonstrated analytical sensitivity comparable to or improved over the initial H3 assay, with a limit of detection of approximately 10^1.7^–10^2.1^ EID_50_/mL and five copies of RNA per reaction. Improved performance was observed for viruses containing A21G with T23G, T23C, or T23A double mismatches in the reverse primer, with a measurable Ct value shift between the initial H3 and H3_v2 assay. The assay showed 100% agreement with expected results in 139 clinical specimens, and no cross-reactivity with other influenza subtypes or other respiratory pathogens. These findings indicate the updated H3_v2 assay improves sensitivity for contemporary and emerging seasonal A(H3N2) viruses while maintaining high specificity. This update ensures continued reliable detection of seasonal A(H3N2), including genetically drifted A(H3N2) viruses, such as the recently emerged A(H3N2) subclade K viruses, and supports global influenza surveillance and diagnostic preparedness.

**IMPORTANCE:**

Accurate detection and subtyping of influenza A(H3N2) viruses are essential for clinical diagnostics, outbreak response, and global influenza surveillance. The emergence and fixation of mutations leading to primer mismatches in the FDA-cleared CDC A(H3) real-time RT-PCR assay raised concerns about reduced sensitivity for contemporary circulating A(H3N2) viruses. To address this, we redesigned the reverse primer and optimized additional assay components to improve detection of currently circulating seasonal A(H3N2) viruses. The updated H3_v2 assay restores and enhances sensitivity, including for viruses harboring key reverse primer mismatches, while maintaining high specificity and complete agreement with clinical specimens. This update ensures reliable detection of seasonal influenza A(H3N2) and A(H3N2) variant viruses, including recently emerged A(H3N2) subclade K viruses, and strengthens diagnostic preparedness and surveillance capacity nationally and internationally.

## INTRODUCTION

Influenza A(H3N2) virus emerged in the human population in 1968 and is one of the three main types of influenza viruses that circulate globally, contributing substantially to annual respiratory illness. Compared with other seasonal influenza virus subtypes or lineages, A(H3N2) viruses evolve rapidly and are frequently associated with more severe epidemics (1–3). In addition to their seasonal burden, A(H3N2) viruses demonstrate the capacity for bidirectional transmission between humans and swine, creating expanded opportunities for viral evolution and zoonotic spillover (4–6).

Influenza surveillance is critical for understanding currently circulating influenza viruses and remaining vigilant for novel influenza viruses. To support national diagnostic capacity, the Centers for Disease Control and Prevention (CDC) distributes an *in vitro* diagnostic (IVD) panel of single-plex real-time reverse transcription-polymerase chain reaction (rRT-PCR) assays that are U.S. Food and Drug Administration (FDA)-cleared for detection and typing/subtyping influenza viruses [510(k) K200370] (7). The CDC Human Influenza Virus Real-Time RT-PCR Diagnostic Panel (CDC Flu rRT-PCR Dx Panel) influenza A subtyping kit is a critical component of this IVD panel and is used to detect and distinguish seasonal 2009 pandemic influenza A(H1N1) [A(H1N1)pdm09] and influenza A(H3N2) subtypes.

The influenza A subtyping kit contains five molecular targets: an influenza A assay (InfA) that targets a region in the matrix (M) gene segment for universal detection of all influenza A viruses; A(H3) and A(H1)pdm09 assays that target the highly conserved region of the hemagglutinin (HA) gene segment to subtype viruses as A(H3N2) or A(H1N1)pdm09; and a pandemic influenza A (pdmInfA) assay that targets the A(H1N1)pdm09 influenza nucleoprotein (NP) gene segment, which can help to identify swine-origin variant influenza viruses. Finally, the kit contains a human specimen control assay that targets the human ribonuclease P/MRP subunit P30 (RP) gene to confirm the presence of human clinical material in samples tested and ensure specimen integrity (7).

In 2020, a clade of influenza A(H3N2), 3C.2a1b.2a, emerged in the HA gene segment containing an A21G mismatch in the reverse primer target region of the H3 assay (H3 IVD) (8). This mismatch was located three nucleotides from the 3′ end of the reverse primer, and the location of this mismatch impacted sensitivity of the H3 component to detect A(H3N2) viruses that were close to the limit of detection (LoD) of the assay. By the end of 2021, this mutation was found within 96% of the globally circulating A(H3N2) viruses based on nucleotide sequence data available from the Global Initiative on Sharing All Influenza Data (GISAID) database (9). We also found viruses with this mismatch and additional mismatches, including a T23G mismatch at the 3′ base of the reverse primer. We updated the design of the H3 assay to shift the reverse primer to a more highly conserved area of the HA gene segment closer to the probe. Minor updates were also made to the forward primer and probe sequence to include mixed bases at known mismatched positions. This updated design restores the sensitivity of the H3 assay and helps to prevent future sensitivity issues as influenza A(H3N2) viruses continue to evolve through the addition of mixed bases to the forward primer and probe sequences.

## MATERIALS AND METHODS

### Influenza viruses

Influenza viruses were grown to high titer in either Madin-Darby Canine Kidney cells or embryonated chicken eggs, and infectious virus titers were measured in the cell culture supernatant or allantoic fluid using tissue culture infectious dose-50% (TCID_50_/mL) or egg-infectious dose-50% (EID_50_/mL) (10). Total nucleic acids were extracted using the Qiagen EZ1 DSP Virus Kit on the Qiagen EZ1 Advanced XL automated extractor (Qiagen, Hilden, Germany; https://www.qiagen.com/us ).

### H3 assay updated primers and probe

The H3_v2 assay adds mixed bases into the forward primer and probe sequences based on known mismatches in the most recent circulating strains and shifts the reverse primer to a more highly conserved region closer to the H3 probe (Table 1; Fig. S1). We used PrimerExpress 3.0.1 software (Applied Biosystems, Foster City, USA) to design the primers/probes to have annealing temperatures of approximately 60°C and 70°C, respectively. We confirmed primer/probe specificity through comparisons with sequence data collected from GISAID between 2020 and 2023.

**TABLE 1:** CDC H3 and H3_v2 component or assay real-time RT-PCR primer and probe sequences

Primer/probe	Sequence 5′−3′	Nucleotide position^*a*^
H3 forward primer	AAG CAT TCC CAA TGA CAA ACC	906-926
H3_v2 forward primer	AAG CAT TCC YAA TGA CAA ACC	
H3 reverse primer	ATT GCR CCR AAT ATG CCT CTA GT	1052-1030
H3_v2 reverse primer	CAT YCC TGT TGC CAA TTT CAG	1008-988
H3 probe	CAG GAT CAC /ZEN/ATA TGG GSC CTG TCC CAG^*b*^	942-968
H3_v2 probe (ZEN)	CAG GAT CAC /ZEN/ATA YGG GGC MTG TCC CAG^*b*^	
H3_v2 probe (BHQ)	CAG GAT CAC[Nova]ATA YGG GGC MTG TCC CAG^*c*^	

^*a*^ Nucleotide positions are according to the HA gene of A/Darwin/9/2021_H3N2 (EPI_ISL_1888006).

^*b*^ Probes are labeled at the 5′ end with the reporter 6-carboxyfluorescein (FAM), a ZEN Quencher between base residues 9 and 10, and an Iowa Black FQ (IBFQ, IWBkFQ) quencher at the 3′ end (Integrated DNA Technologies, Coralville, IA).

^*c*^ Probes are labeled at the 5′ end with the reporter molecule 6-carboxyfluorescein (FAM), a "nova" quencher between base residues 9 and 10, and a Black Hole Quencher 1 (BHQ-1) at the 3′ end (Biosearch Technologies, Inc., Novato, CA).

Two types of TaqMan hydrolysis probes, the BHQnova double-quenched (BHQ) probe and the ZEN double-quenched (ZEN) probe have been evaluated. The reporter molecule 6-carboxyfluorescein (FAM) was used to label both probes at their 5' ends. The BHQ probe and ZEN probe each have a Black Hole Quencher (BHQ) or an Iowa Black FQ at their 3' ends, as well as an internal nova (BHQ probe) or ZEN (ZEN probe) quencher incorporated between base residues 9 and 10 (Table 1).

The H3_v2 primers and dual-labeled TaqMan hydrolysis probes were synthesized by Biosearch Technologies, Inc. (Petaluma, CA, USA) and Integrated DNA Technologies, Inc. (Coralville, IA, U.S.)

### rRT-PCR reaction conditions

Reaction conditions for the H3_v2 assay were based upon the FDA–cleared CDC Flu rRT-PCR Dx Panel. PCR reactions were performed using Invitrogen SuperScript III Platinum One-Step quantitative RT-PCR on the Applied Biosystems (AB) 7500 Fast Dx Real-Time PCR instrument (Life Technologies). All rRT-PCR reactions performed had a total reaction volume of 25 µL with primer and probe reaction concentrations at 0.8 µM and 0.2 µM, respectively. Thermocycling rRT-PCR conditions were as follows: 50°C for 30 min, *Taq* activation at 95°C for 2 min, and 45 cycles of 95°C for 15 s and 55°C for 30 s. A thermal gradient analysis confirmed comparable performance within the annealing temperature range from 51°C–62.5°C; therefore, we have chosen to maintain the annealing temperature at 55°C (Table S1, Fig. S2) (7, 11).

All analytical performance data were collected on an ABI 7500 Fast Dx Real-time PCR instrument. Fluorescent signal was captured during the annealing step of the reaction. All data were analyzed with the sequence detector software v1.4.1 (Life Technologies).

### Analytical sensitivity and specificity

The analytical sensitivity of the H3_v2 assay was determined using two titered A(H3N2) virus strains, A/Darwin/09/2021 (clade 3C.2a1b; GISAID accession no. EPI2415934), which contains the A21G mismatch in the reverse primer region, and A/Hong Kong/4801/2014 (clade 3C.2a; EPI614437), which does not contain the A21G mismatch in the reverse primer region. In addition, Twist synthetic RNA material contained the HA gene sequence derived from the A/Darwin/09/2021_A(H3N2) strain (EPI 1888006) was included (Twist Bioscience, https://www.twistbioscience.com/). The evaluation was conducted by comparisons with the performance of the H3 assay from the CDC Flu rRT-PCR Dx Panel.

The assay specificity (inclusivity and exclusivity) of the H3_v2 assay was evaluated using ZEN probe chemistry against a panel of influenza A viruses (Tables 4 and 5; Table S3). The panel contained nine A(H3N2) strains, including one A(H3N2) subclade K virus that has become dominant among circulating A(H3N2) viruses during the 2025/26 season (12, 13). It also contained three A(H1N1)pdm09 strains and six avian influenza A viruses, including A(H5N8), A(H7N9), and other subtypes. In addition, the panel included A(H1)/A(H3) variant [A(H1)v/A(H3)v] viruses that typically circulate in swine and have caused outbreaks of illness and pose a continuing pandemic threat to human populations (5). Three distinct A(H3)v variant clades (2010.1, 1990.1, and 1990.4) were evaluated. A collection of influenza B and C viruses, coronaviruses, and human non-influenza respiratory pathogens was also used to test the analytical specificity (exclusivity) of the H3_v2 assay.

### Assay performance with clinical specimens

The clinical performance of the H3_v2 assay was evaluated using a panel of 79 residual A(H3N2) clinical specimens, including 19 A(H3N2) subclade K specimens, all of which had associated next-generation sequencing data. Among the 60 non-subclade K H3-positive specimens collected in 2022, 30 contained the A21G mismatch in the initial H3 reverse primer binding site, with one of these specimens also showing an additional T23G mismatch. Of the 19 subclade K specimens collected in 2025, 14 contained A21G and T23C double mismatches, and five contained A21G and T23A double mismatches in the reverse primer binding site. The other 30 specimens had no mismatches in the target region. The 60 specimens collected between 2019 and 2021 that were negative by the H3 assay were also tested. The 139 upper respiratory specimens consisted of nasal swabs, throat swabs, and nasopharyngeal swabs collected from multiple surveillance sites. Total nucleic acids from 120 µL of each clinical specimen were extracted using the Qiagen EZ1 DSP Virus Kit on the Qiagen EZ1 Advanced XL automated extractor (Qiagen, Hilden, Germany; https://www.qiagen.com/us). Extracted material was eluted in 120 µL of elution buffer. All RNA samples used in this study had undergone one freeze-thaw cycle before testing. Specimens were tested with the initial H3 assay, the H3_v2 assay (both manufacturers), and the RP assay. Analysis settings were identical across all clinical specimens tested.

## RESULTS

### H3_v2 assay update

The H3_v2 rRT-PCR assay was developed using the ABI 7500 Fast Dx Real-time PCR system for the qualitative detection and characterization of influenza A(H3N2) virus RNA in respiratory specimens from patients presenting with influenza-like illness and/or acute respiratory illness. Both the forward primer and probe of the H3_v2 assay were developed from the initial H3 assay, with minor adjustments. These included updating the T base to the degenerate base Y (C + T) at position 13 (T13Y) in the probe, as the T13C mismatch has been observed in over 99% of circulating seasonal A(H3N2) viruses based on sequence data available from GISAID. Similarly, the forward primer was updated from C to Y at position 13 (C13Y) due to mismatches with recently circulating viruses. The reverse primer was newly designed to target a more conserved region and address the A21G mismatch within the initial H3 assay, which has increased to more than 99% as of June 2026, and has also resulted in two mismatches, specifically A21G with T23C (85.27%), T23A (0.95%), or T23G (0.03%) as of June 2026 (Table S1). According to the thermal gradient analysis of two types of TaqMan hydrolysis probes with two virus strains, we maintained the annealing temperature at 55°C (Table S2; Fig. S2). This temperature is consistent with the initial CDC Flu rRT-PCR Dx Panel and effectively accommodates potential nucleotide mismatches in the primer and probe regions due to the genetic variability of the viruses.

### Analytical sensitivity

The LoD of the H3_v2 assay was determined by analyzing five-fold serial dilutions of two A(H3N2) virus strains, A/Darwin/09/2021 and A/Hong Kong/4801/2014. The five-fold serial dilutions of these representative strains were tested in triplicate with both the H3 and the H3_v2 assays to determine the preliminary LoD. Once the range was established, the LoD was determined (*N* = 20) for each virus. Data shown in Table 2 indicate the mean Ct values at each dilution for each probe. The LoD of the H3_v2 assay was calculated to indicate the lowest detectable concentration of influenza virus (EID_50_/mL) with a 95.0% or greater positivity. Using the results of the higher virus concentration of the H3_v2 assay, the LoD for each primer and probe set was determined to be 10^1.66–2.12^ EID_50_/mL. This correlates to 10^-0.64~ -0.18^ EID_50_ per reaction (5.0 µL/reaction) (Table 2; Table S3 and Fig. S3). Similarly, we assessed copy number sensitivity by using a serial dilution of a synthetic A(H3N2) HA gene RNA (GISAID accession no. EPI_ISL_1888006; Twist Bioscience). The H3_v2 assay could detect as few as five copies per reaction (Table 3).

**TABLE 2:** Assay limit of detection with influenza A(H3N2) virus strains (*N* = 20)^*b*^

Infectious titer^*a*^	Ct values of H3		Ct values of H3_v2			
	Positive (%)	Mean ± SD	ZEN probe		BHQ probe	
			Positive (%)	Mean ± SD	Positive (%)	Mean ± SD
A/Darwin/9/2021 (clade 3C.2a1b/EPI2415934/containing A21G mismatch in H3 reverse primer)						
10 ^2.13^	100	32.47 ± 0.71	100	32.61 ± 0.47	100	33.47 ± 0.41
**10** ^**1.66*b***^	100	34.59 ± 1.00	100	34.43 ± 0.64	100	35.40 ± 0.62
A/Hong Kong/4801/2014 (clade 3C.2a/EPI614437/no A21G mismatch in H3 reverse primer)						
10 ^2.60^	100	32.42 ± 0.42	100	31.30 ± 0.82	100	33.20 ± 0.72
**10** ^**2.12*b***^	100	34.26 ± 0.98	100	33.20 ± 0.91	100	34.77 ± 0.81

^*a*^ Data represent EID_50_/mL.

^*b*^ The infectious titers, highlighted in bold, indicated the limit of detection of H3_v2 assay for each virus strain.

**TABLE 3:** Assay limit of detection determined using a quantified synthetic RNA^*a*^

No. of RNA copies/reaction	Ct values of H3_v2							
	ZEN probe			Mean ± SD	BHQ probe			Mean ± SD
50,000	17.79	17.68	17.62	17.70 ± 0.09	18.79	18.47	18.95	18.73 ± 0.24
5,000	20.99	21.14	21.01	21.04 ± 0.08	21.94	21.81	22.67	22.14 ± 0.47
500	24.40	24.35	24.43	24.39 ± 0.04	25.23	25.15	25.34	25.24 ± 0.09
50	27.76	27.75	27.80	27.77 ± 0.03	28.59	28.48	28.59	28.56 ± 0.06
5	31.08	30.86	31.18	31.04 ± 0.16	32.05	33.52	33.18	32.92 ± 0.77

^*a*^ The quantified RNA material received from Twist Bioscience (https://www.twistbioscience.com/) included the HA gene of influenza A (H3N2) virus strain A/Darwin/09/2021 (GISAID accession no. EPI1888006).

The Ct cutoff value for the H3 and H3_v2 assays was established as <38 for domestic human diagnostic testing under FDA 510(k) clearance in the United States. This cutoff was based on the LoD studies and is consistent with the Ct cutoff (<38) previously established for the CDC Flu rRT-PCR Dx Panel (14).

### Analytical specificity

Initially, the H3_v2 design was screened using no-template control reactions; we found no intramolecular or intermolecular nonspecific interactions that resulted in any products (data not shown).

To evaluate assay specificity (inclusivity), two dilutions of 14 influenza A(H3N2) viruses were tested in triplicate, including nine seasonal A(H3N2) strains collected between 2009 and 2025 (including one subclade K strain) and five A(H3)v strains. Across all nine A(H3N2) viruses, the H3_v2 assay demonstrated comparable or improved performance relative to the initial H3 assay using ZEN probe chemistry. Notably, A/Massachusetts/10/2021, which contains A21G and T23G double mismatches in the reverse primer binding region, showed marked improvement with H3_v2, exhibiting a reduction of more than ten Ct values compared with the initial H3 assay. Similarly, the subclade reference K virus, A/Wisconsin/114/2025 (EPI4617035), harboring A21G and T23C double mismatches in the reverse primer, demonstrated a comparable substantial improvement relative to the initial H3 assay. The five A(H3)v strains representing three distinct swine-origin variant clades (2010.1, 1990.1, and 1990.4) also showed comparable or enhanced detection performance with the H3_v2 assay, supporting its broad inclusivity across contemporary seasonal A(H3N2) and A(H3)v viruses (Table 4).

**TABLE 4:** Assay specificity (inclusivity) evaluation with influenza A(H3N2) and A(H3)v clade viruses^*a*^

Influenza A(H3N2) virus	H3 or H3v clade	GISAID accession no.	EID_50_/mL	Ct values of rRT-PCR					
				H3			H3_v2 (ZEN probe)		
A(H3N2) virus									
A/Wisconsin/114/2025	subclade	EPI_ISL_20131564	not available	0	0	0	**29.75**	**29.97**	**29.57**
				40.23	39.58	0.00	**26.02**	**26.01**	**25.49**
A/Massachusetts/10/2021	3C.2a1b	EPI_ISL_8557874	10 ^0.8^	0	0	0	**30.43**	**30.68**	**30.71**
			10 ^1.8^	42.19	38.26	38.73	**26.46**	**26.64**	**26.58**
A/Darwin/9/2021	3C.2a1b	EPI_ISL_16998754	10 ^2.61^	33.78	33.79	33.42	31.61	30.99	31.60
			10 ^3.57^	29.91	30.21	30.10	27.13	27.61	28.04
A/Cambodia/e0826360/2020	3C.2a1b	EPI1853346	10 ^2.2^	40.16	39.87	38.47	34.07	33.51	34.53
			10 ^3.2^	35.16	35.45	35.73	29.91	29.89	30.02
A/Hong Kong/2671/2019	3C.2a1b	EPI1592035	10 ^2.5^	38.43	39.05	37.57	33.97	34.10	35.18
			10 ^3.5^	37.30	36.34	36.54	31.16	30.99	31.37
A/Singapore/INFIMH-16-0019/2016	3C.2a1	EPI_ISL_291269	10 ^2.5^	33.06	32.39	32.58	34.83	34.24	34.37
			10 ^3.5^	29.31	29.47	29.08	30.11	30.53	30.81
A/Hong Kong/4801/2014	3C.2a	EPI_ISL_17668130	10 ^2.61^	32.57	32.10	32.68	31.98	32.33	32.30
			10 ^4.05^	27.04	27.16	27.21	25.78	25.74	26.07
A/Texas/50/2012	3C.1	EPI_ISL_129858	10 ^2.5^	34.88	36.20	34.81	36.52	35.62	0.00
			10 ^3.5^	32.67	32.36	31.20	33.21	33.24	34.19
A/Perth/16/2009	PERTH16-like	EPI_ISL_31913	10 ^2.3^	38.10	37.03	37.77	33.14	33.44	33.29
			10 ^3.3^	33.45	33.65	33.53	29.55	29.82	29.63
A(H3N2) variant virus									
A/New Mexico/19/2022	H3v 2010.1	EPI_ISL_15619273	10 ^2.16^	40.51	40.47	41.24	34.23	33.82	34.23
			10 ^3.16^	35.36	35.60	35.79	31.50	31.26	31.08
A/Ohio/13/2017	H3v 2010.1	EPI_ISL_277234	10 ^2.6^	34.97	35.20	34.70	29.86	29.65	29.59
			10 ^3.6^	30.63	31.04	30.96	26.15	26.08	26.14
A/Michigan/84/2016	H3v 1990.4	EPI_ISL_232047	10 ^2.5^	0	0	43.64	41.58	38.64	38.86
			10 ^3.5^	36.23	39.39	39.86	35.13	35.38	35.10
A/West Virginia/06/2011	H3v 1990.4	EPI90862	10 ^2.9^	35.54	35.19	35.25	32.01	31.76	31.67
			10 ^3.9^	31.22	31.40	31.28	28.30	28.06	28.11
A/Hawaii/28/2020	H3v 1990.1	EPI1766950	10 ^4.53^	37.62	37.27	37.49	34.32	34.68	34.92
			10 ^5.53^	33.29	33.55	33.33	31.35	31.55	31.63

^*a*^ Ct value of 0 means that no amplification was observed: Ct values for the H3_v2 assay that improved by more than seven cycles compared with the H3 assay are highlighted in bold.

To demonstrate that the H3_v2 assay retains specificity (exclusivity) against non-A(H3N2) influenza viruses and other respiratory pathogens, we tested a panel of common respiratory pathogens and influenza viruses considered genetic near neighbors. Nucleic acids from high-titer viral preparations were extracted and tested with the H3_v2 assay. As expected, all non-A(H3N2) influenza viruses, including three each of A(H1N1)pdm09 strains and A(H1)v strain and two influenza B viruses, tested negative with the H3_v2 assay while remaining positive with the InfA or influenza B (InfB) assays, respectively (Table 5). No cross-reactivity was observed with three influenza C viruses or with 39 non-influenza respiratory pathogens (20 viruses and 19 bacteria) (Table 5; Table S4). These results demonstrate that the H3_v2 assay is specific to the detection of influenza A(H3N2) and A(H3)v viruses without cross-reactivity to other respiratory pathogens or genetically related influenza viruses, including severe acute respiratory syndrome coronavirus 2 (SARS-CoV-2).

**TABLE 5:** Assay specificity (exclusivity) evaluation with non–A(H3N2) influenza viruses

Influenza viruses	Subtype/Lineage	ID50/mL	rRT-PCR result	
			InfA/InfB	H3_v2 (ZEN probe)^*b*^
A/BRISBANE/02/2018	A(H1N1)pdm09	10 ^7.9^	+/−	−
A/MICHIGAN/45/2015	A(H1N1)pdm09	10 ^8.3^	+/−	−
A/CALIFORNIA/08/2009	A(H1N1)pdm09	10 ^9.2^	+/−	−
A/OHIO/24/2017	A(H1N2)v_1A.1.1	10 ^6.3^	+/−	−
A/OHIO/09/2015	A(H1N1)v_1A.3	10 ^7.7^	+/−	−
A/OHIO/35/2017	A(H1N2)v_1B.2.1	10 ^6.9^	+/−	−
A/CANINE/FLORIDA/43/2004	A(H3N8)	10 ^8.1^	+/−	−
A/EQUINE/OHIO/01/2003	A(H3N8)	10 ^8.4^	+/−	−
A/ASTRAKHAN/3212/2020 IDCDC-RG71A	A(H5N8)	10 ^8.7^	+/−	−
A/GYRFALCON/WASHINGTON/41088-6/2014	A(H5N8)	10 ^8.9^	+/−	−
A/TAIWAN/1/2017	A(H7N9)	10 ^5.5^	+/−	−
A/BANGLADESH/0994/2011	A(H9N2)	10 ^9.5^	+/−	−
B/WASHINGTON/02/2019	B(Victoria)	10 ^9.3^	−/+	−
B/PHUKET/3073/2013	B(Yamaga)	10 ^9.9^	−/+	−
C/Minnesota/1/2016	C(Kanagawa176)	NA^*a*^	−/−	−
C/Minnesota/4/2015	C(Kanagawa176)	NA	−/−	−
C/Minnesota/29/2015	C(Sao Paulo82)	NA	−/−	−

^*a*^ NA, not available.

^*b*^ +, detected; −, not detected.

Similar results were also obtained for assay specificity when using the BHQ probe (data not shown).

### Performance with clinical specimens

We evaluated the clinical performance of the H3_v2 assay by testing 139 residual clinical specimens, including 79 H3-positive clinical specimens with next-generation sequencing confirmation of the presence of seasonal influenza A(H3N2) virus. In our evaluation of the H3_v2 assay, we found that 29 H3-positive specimens (S1–S29) with the A21G mismatch only in the H3 reverse primer, along with 30 H3-positive specimens (S30–S59) without this mismatch, demonstrated 100% agreement with the expected values for each specimen, even the specimens with Ct values greater than 30 (three specimens with mismatches and eight specimens without mismatches) (Table 6; Table S5). In addition, one non-subclade K specimen (S60) containing A21G and T23G double mismatches and 19 subclade K specimens (S61–S79), including 14 with A21G and T23C and five with A21G and T23A double mismatches in the reverse primer exhibited a significant improvement in the performance of the H3_v2 assay compared to the H3 assay, showing a difference of more than seven Ct values (Table 7). As expected, all 60 H3-negative specimens were also negative in the H3_v2 and InfA assays (data not shown).

**TABLE 6:** Clinical performance of H3_v2 real-time RT-PCR assay with residual clinical specimens

Performance measure		Next-generation sequencing result			
		Positive	Negative	Total	
H3_v2 rRT-PCR result	Positive	79/79	0/0	79/79	Sensitivity
					100.00%
	Negative	0/0	60/60	60/60	Specificity
					100.00%
	Total	79	60	139/139	Positive percent agreement
					100.00%

**TABLE 7:** Clinical specimen evaluation with influenza A(H3N2) residual clinical specimens containing double mismatches in the H3 reverse primer region^*a*^^,^^*b*^

Specimen no.	H3 clade	GISAID accession no.	Mismatches in reverse primer	Ct values of rRT-PCR		
				H3	H3_v2	
					ZEN probe	BHQ probe
S60	3C.2a1b	EPI3351361	A21G + T23G	31.87	**23.62**	**23.25**
S61	Subclade K	EPI5301128	A21G + T23C	0	**29.09**	**32.70**
S62	Subclade K	EPI5301010	A21G + T23C	35.22	**24.99**	**27.29**
S63	Subclade K	EPI5300986	A21G + T23C	0.00	**28.86**	**31.86**
S64	Subclade K	EPI5301176	A21G + T23C	0.00	**31.49**	**32.13**
S65	Subclade K	EPI5301205	A21G + T23C	35.91	**24.86**	**28.23**
S66	Subclade K	EPI5301152	A21G + T23C	0	**28.86**	**32.13**
S67	Subclade K	EPI5301080	A21G + T23C	35.94	**27.45**	30.83
S68	Subclade K	EPI5301104	A21G + T23C	37.75	**27.14**	**30.12**
S69	Subclade K	EPI5301034	A21G + T23C	35.17	**24.42**	**27.24**
S70	Subclade K	EPI5192742	A21G + T23C	0.00	**33.43**	**37.35**
S71	Subclade K	EPI5192798	A21G + T23C	30.86	**19.62**	**21.86**
S72	Subclade K	EPI5192734	A21G + T23C	35.73	**24.39**	**27.50**
S73	Subclade K	EPI5192774	A21G + T23C	0	**31.42**	**33.59**
S74	Subclade K	EPI5192710	A21G + T23C	34.69	**24.18**	**26.98**
S75	Subclade K	EPI5300938	A21G + T23A	41.85	**28.61**	**31.85**
S76	Subclade K	EPI5192766	A21G + T23A	35.78	**22.03**	**25.42**
S77	Subclade K	EPI5192718	A21G + T23A	0	**23.59**	**26.54**
S78	Subclade K	EPI5192790	A21G + T23A	36.92	**25.03**	**27.63**
S79	Subclade K	EPI5192686	A21G + T23A	35.08	**22.97**	**25.53**

^*a*^ The clinical specimen evaluation along with the remaining influenza A(H3N2) residual clinical specimens on Table S5.

^*b*^ Ct value of 0 means that no amplification was observed; Ct values for the H3_v2 assay that improved by more than seven cycles compared with the H3 assay are highlighted in bold.

### *In-silico* analysis of primers and probes mismatches among A(H3N2) subclade K viruses

To assess potential mismatches between the HA gene sequences and the H3 and H3_v2 primers and probe, an *in-silico* analysis was performed using 35,400 HA gene sequences of A(H3N2) subclade K viruses deposited in GISAID from 1 August 2025 to 30 June 2026.

The percentages of sequences with no mismatch in the H3_v2 forward primer, reverse primer, and probe regions were 94.99%, 97.38%, and 98.23%, respectively; very few viruses contained two or three mismatches in the primer or probe region. However, a single mismatch in the probe binding region was observed in 96.42% of sequences, and double mismatches in the reverse primer binding region were observed in 97.51% of sequences. These mismatches warrant further evaluation to determine their potential impact on assay performance. (Table 8)

**TABLE 8:** *In silico* analysis of H3 and H3_v2 primer and probe mismatches among influenza A(H3N2) subclade K viruses^*a*^

Primer and probe	Number(%) of mismatched nucleotides					Total
	0	1	2	3	≥4	
H3/H3_v2 Forward	33,626 (94.99)	1,764 (4.98)	10 (0.01)	0 (0)	0 (0)	35,400
H3_v2 Probe	34,471 (97.38)	911 (2.57)	18 (0.05)	0 (0)	0 (0)	35,400
H3_v2 Reverse	34,772 (98.23)	621 (1.75)	7 (0.02)	0 (0)	0 (0)	35,400
H3 Probe	380 (1.07)	34,134 (96.42)	867 (2.45)	19 (0.05)	0 (0)	35,400
H3 Reverse	27 (0.08)	620 (1.75)	34,519 (97.51)	232 (0.66)	2 (0)	35,400

^*a*^ The HA gene sequences deposited in GISAID between 1 August 2025, and 30 June 2026.

## DISCUSSION

The analytical sensitivity demonstrated the H3_v2 assay was comparable to the initial H3 assay prior to the emergence of mismatched viruses. The H3_v2 assay detected virus titers as low as 10^1.66^–10^2.12^ EID 50/mL (or 10^-0.64^–10^−0.18^ EID50/reaction) of influenza A(H3N2) viruses. The updated assay also demonstrated robust performance across a dynamic range of quantified synthetic RNA and detected as few as five RNA copies per PCR reaction.

The assay specificity (inclusivity) evaluation demonstrated that the H3_v2 assay was also comparable to or improved to the initial H3 assay for detection of A(H3N2) viruses, including subclade K viruses. The viruses that have A21G and T23C, T23A or T23G double mismatches in the H3 reverse primer binding region showed markedly improved detection by H3_v2 compared with the initial H3 assay. The A(H3)v viruses that tested positive in the InfA and pdmInfA components using the CDC influenza A subtyping kit also showed comparable or improved performance relative to the H3 component targeting the HA gene segment. Importantly, our data demonstrate that the H3_v2 assay is specific to A(H3N2) and A(H3)v viruses, as we observed no cross-reactivity with other viruses tested in our exclusivity evaluation, including high-titer concentrations of seasonal A(H1N1)pdm09 or influenza B lineages, as well as A(H5) and A(H7N9) viruses and other common respiratory viruses, including SARS-CoV-2.

The performance evaluation of clinical specimens further confirmed H3_v2 assay’s high sensitivity and specificity for detecting A(H3N2) viruses. 20 H3-positive clinical specimens, including 19 subclade K specimens, containing A21G with T23G, T23C, or T23A double mismatches showed a dramatic Ct shift between the initial H3 assay and the H3_v2 assay, highlighting reduced sensitivity of the initial H3 assay when multiple mismatches occur within the reverse primer region.

Sequence analysis of HA gene segments from A(H3N2) viruses indicated that the A21G mismatch in the H3 reverse primer binding region has exceeded 96% prevalence since 2021. While a single A21G mismatch did not substantially affect assay performance, reduced sensitivity was observed when A21G occurred in combination with a second mismatch T23G, T23C or T23A. In addition, the prevalence of A21G and T23C mismatches in A(H3N2) HA gene segments has increased to >85% by the mid-2026(Table S1). Together with observed reduced sensitivity of the H3 assay for viruses/specimens containing two mismatches within the reverse primer region, these findings indicate that an assay update is necessary to ensure comprehensive detection of circulating A(H3N2) viruses.

Although nearly 99% of human seasonal A(H3N2) viruses contain a T13C mismatch, the probe position was updated from T to Y in H3_v2 to maintain inclusivity for A(H3)v viruses, which consistently encode T at this position (Table 1).

Throughout the evaluation, the H3_v2 assay performed equivalently using both BHQ and ZEN double-quenched probe chemistries. Additionally, the evaluation using the Quanta BioSciences qScript One-Step qRT-PCR kit, Low ROX (Quanta BioSciences 95059-200), produced results comparable to the Invitrogen SuperScript III Platinum One-Step kit (Data not shown) (15)

Since August 2025, there has been a rapid increase in A(H3N2) subclade K (formerly J.2.4.1) viruses detected from several countries based on available genetic sequence data. This subclade contains multiple HA gene amino acid substitutions compared to related A(H3N2) viruses and has become dominant during the 2025/26 season, replacing other A(H3N2) strains in a manner consistent with a transmission fitness advantage (12, 13, 16). Sequence analysis revealed that all viruses containing double mismatches (A21G in combination with T23C or T23G) within the H3 reverse primer-binding region belonged to subclade K (Table S1). *In silico* analysis indicated that H3_v2 does not exhibit concerning mismatch patterns with subclade K sequences, and testing of one cultured subclade K virus and 20 subclade K clinical specimens demonstrated improved sensitivity compared to the H3 assay.

Influenza viruses evolve rapidly and can generate genetic diversity through mutation and reassortment. Therefore, it is critical to continuously monitor assay performance to assess the potential effects of genetic changes that may arise through mutation or reassortment and to update the assay as needed in the future. Ensuring that these assays have high sensitivity and specificity to detect circulating flu viruses is critical for ongoing influenza surveillance activities and downstream viral characterization, which ultimately supports seasonal vaccine strain selection.

The H3 assay, developed and FDA-cleared in 2008, provided rapid, sensitive, and specific detection of seasonal influenza A(H3N2) viruses and A(H3)v viruses for over 16 years (14). The updated version of the assay, H3_v2, received FDA clearance in 2024 and improves sensitivity and maintains specificity for surveillance and diagnostic applications for seasonal influenza A(H3N2) viruses and A(H3)v viruses, including detection of recently emerged A(H3N2) subclade K strains (Fig. S4) (15, 17). Since March 2025, the CDC Influenza A Subtyping Kit (Ver 4), which contained the H3_v2 primers and probe, has been deployed to Public Health Laboratories. The updated assay will continue to support domestic and global influenza surveillance and diagnostic preparedness.

## SUPPLEMENTAL MATERIAL

Supplemental tables and figures jcm.00727-26-s0001.docx Tables S1 to S5 and Fig. S1 to S4.

ASM does not own the copyrights to Supplemental Material that may be linked to, or accessed through, an article. The authors have granted ASM a non-exclusive, world-wide license to publish the Supplemental Material files. Please contact the corresponding author directly for reuse.
