# Supplementary material for: Update to the CDC influenza A(H3) subtyping assay for improved detection of influenza A(H3N2) viruses, including subclade K viruses
Source: J Clin Microbiol. 2026 Oct 27;64(11):e00727-26. doi: 10.1128/jcm.00727-26 (PMC13753822; doi:10.1128/jcm.00727-26)
Supplement: Supplementary file 1 [file jcm.00727-26-s0001.docx]

Table S1. *In silico* analysis of H3 and H3_v2 primer and probe mismatches among influenza A(H3N2) viruses

The HA gene sequences deposited in GISAID between August 1^st^, 2025, and June 30^th^, 2026.

V=A, C and G

Table S2. Thermal gradient analysis with influenza A(H3N2) viruses

 Thermal gradient analysis was performed on a Bio-Rad CFX96™ Real-Time PCR Detection System

* relative fluorescence units

Table S3. Range finding of assay sensitivity with influenza A(H3N2) viruses (N=3)

Note: CT value of 0 means that no amplification was observed.

Table S4. Assay specificity (exclusivity) evaluation with human non-influenza respiratory pathogens Table S4a. Respiratory viruses

Table S4b. Respiratory bacterica

Table S5. Clinical specimen evaluation with influenza A(H3N2) residual clinical specimens containing A21G only or no mismatch in the H3 reverse primer region

29 specimens (S1-S29) were with the A21G mismatch only in the H3 reverse primer region; 30 specimen (S30-S59) were without any mismatch in the H3 reverse primer region.

Figure S1**. Sequence alignment of H3 and H3_v2 assays with influenza A(H3N2) vaccine viruses recommended by WHO from 2002-2026**

Figure S2**.** Amplification chart of thermal gradient analysis of H3_v2 rRT-PCR assay

Thermal gradient analysis was performed using viral RNA of two A(H3N2) viruses with annealing temperatures ranging from 50.0-65.0°C.

2A: A/Darwin/09/2021 with ZEN^TM^ Double-Quenched probe

2B: A/Darwin/09/2021 with BHQnova™ double-quenched probe

2C: A/Hong Kong/4801/2014 with ZEN^TM^ Double-Quenched probe

2D: A/Hong Kong/4801/2014 with BHQnova™ double-quenched probe

Figure S3. Analytical Sensitivity-Standard Curves

H3 and H3_v2 Standards

Ct values (dR)

| Serial dilutions of RNA extracted from A/Darwin/9/2021 | H3 | H3_v2 |
| --- | --- | --- |
| 10 2.13 | 34.95 | 34.31 |
| 10 2.61 | 33.6633 | 32.2033 |
| 10 3.09 | 31.5067 | 29.5933 |
| 10 3.57 | 30.0733 | 27.71 |
| 10 4.05 | 27.6867 | 25.8633 |

Standard curves of H3 and H3_v2 (ZEN probe) assays were generated using a fivefold serial dilution of RNA extracted from A/Darwin/9/2021 (10^4.05^, 10^3.57^, 10^3.09^. 10^2.61^ and 10^2.13^ EID_50_/mL). The H3_v2 and H3 assays yielded R² values of 0.9958 and 0.9907, respectively, with regression equations of y = −2.1387x + 36.352 and y = −1.8117x + 37.011, respectively.

Figure S4. Workflow Schematic: Assay investigation, Optimization and Regulatory Evaluation
